# Impact of age and treatment-related adverse events on financial toxicity among patients with breast cancer: a systematic review

**DOI:** 10.1007/s11764-025-01881-7

**Published:** 2025-08-30

**Authors:** Sara P. Myers, Claire R. Morton, Paul A. Bain, Samilia Obeng-Gyasi, Electra D. Paskett, Ann S. McAlearney, Rachel A. Greenup, Christina A. Minami, Elizabeth A. Mittendorf, Tari A. King

**Affiliations:** 1 Division of Breast Surgery, Department of Surgery, Brigham and Women’s Hospital, Boston, MA, USA; 2 Breast Oncology Program, Dana-Farber Brigham Cancer Center, Boston, MA, USA; 3 Harvard Medical School, Boston, MA, USA; 4 Department of Surgery, Yale School of Medicine, New Haven, CT, USA; 5 Center for Surgery and Public Health, Brigham and Women’s Hospital, Boston, MA, USA; 6 Countway Library, Harvard Medical School, Boston, MA, USA; 7 Division of Surgical Oncology, Department of Surgery, The Ohio State University, Columbus, OH, USA; 8 Division of Cancer Prevention and Control, Department of Internal Medicine, College of Medicine, The Ohio State University, Columbus, OH, USA; 9 CATALYST, Center for Advancement of Team Science, Systems Thinking in Health Services and Implementation Science Research, College of Medicine, The Ohio State University Analytics, and Columbus, OH, USA; 10 Department of Family and Community Medicine, College of Medicine, The Ohio State University, Columbus, OH, USA

**Keywords:** Financial toxicity, Young adults, Breast cancer, Age, Treatment-related adverse events

## Abstract

**Purpose:**

Increasing incidence of breast cancer among young adults (YA), defined by the National Comprehensive Cancer Network (NCCN) as age < 40 years, has amplified concerns over financial toxicity (FT) in this demographic. This systematic review sought to clarify the association between YA status and FT.

**Methods:**

We queried bibliographic databases for full-text English language publications between 2010 and 2024 reporting FT using validated patient-reported outcome metrics among patients with stage I–III breast cancer. Two reviewers screened and extracted data between June and September 2024. Variables of interest included age and treatment-related adverse events (secondary outcome of interest).

**Results:**

Of 4023 articles screened, 66 were included for review. Sixty-four were excluded for including in situ or metastatic disease, multiple cancer histologies, or FT as a parameter of a larger scale without validation for stand-alone use. While no studies compared FT using the NCCN YA threshold (i.e., < 40 versus ≥ 40 years), two studies demonstrated that younger age was associated with worse FT. One study compared patients age ≥ 65 to < 65 years using the Comprehensive Score for Financial Toxicity, and the other considered age as a continuous variable using the Psychological Sense of Economic Hardship Scale. The latter also reported treatment-related symptoms were associated with greater FT after adjusting for age.

**Conclusion:**

No study identified the multidimensional domain of FT as a primary outcome among YA versus non-YA women with non-metastatic invasive breast cancer. Two studies showed younger age was associated with greater FT.

**Implications for Cancer Survivors:**

As FT has been shown to negatively impact patient outcomes, further investigations are needed to support policy efforts and to improve care for younger breast cancer survivors.

## Introduction

The rising cost of cancer care [1] represents a significant burden both to the U.S. economy and to individual patients contending with treatment-related expenses [2]. Financial toxicity (FT), or the negative impact of medical debt on quality of life [3] and clinical outcomes [4], has become alarmingly prevalent among survivors [5]. Previous studies have shown that young adults (YA), defined as aged < 40 years according to the National Comprehensive Cancer Network (NCCN), are among those at high risk for experiencing financial hardship after their cancer diagnosis [6, 7].

Financial considerations are especially salient in breast cancer. As a leading cause of cancer-associated mortality among women in the U.S. [8], breast cancer is responsible for upwards of $30 billion in national annual expenditures [9]. Cost-sharing can lead to sustained financial difficulty for many of these patients [10–13]. Although breast cancer-specific studies corroborate findings from investigations agnostic to histologic type, i.e., that younger age is associated with greater FT, several factors complicate our understanding about whether these data are valid when designating cohorts by YA status (≤ 39 years versus > 40 years). Despite the NCCN recognizing YAs as a subgroup facing unique challenges deserving of YA-specific programming, the literature pertaining to financial hardship rarely uses this threshold to define young versus older cohorts. Moreover, although many NCCN member institutions now perform screening for FT, few target YAs [14]. As a result, these patients may not be offered resources such as financial navigation, which have been shown to be effective [15].

In light of evidence that breast cancer incidence is increasing among YAs [16], YAs experience worse survival compared to their older counterparts [17], and that FT potentially contributes to poor outcomes [18], a more complete understanding of the association between YA status and financial hardship is needed to inform future policy and interventions. In particular, studies that consider how age-based differences in epidemiology impact cost of care and those that incorporate rigorously validated metrics for FT would improve applicability of data and allow for more accurate comparisons across treatment experiences. Specifically, since a greater proportion of YAs present with advanced disease at presentation and/or aggressive tumor biology [16, 17], investigations that differentiate risk for FT by treatment pathway (e.g., curative intent versus palliation) would be useful in designing feasible strategies to address financial burden. To this end, we performed a systematic review among U.S. women diagnosed with stage I–III breast cancer to understand the relationship between YA status and FT. We hypothesized that YAs would be at higher risk for FT than non-YAs. As treatment-related adverse events may contribute significantly to long-term financial hardship and vary by age [19, 20], a secondary outcome of interest involved exploring how these complications modulate the association between YA and FT.

## Methods

A systematic review of the literature was conducted to understand whether for patients with stage I–III breast cancer age < 40 years compared to age ≥ 40 years impacted treatment-related FT. Secondarily, we sought to explore whether treatment-related complications that may have been discussed in included articles made YAs more susceptible to FT. The conduct of this review was guided by the JBI Manual for Evidence [21] and reported in accordance with the Preferred Reported Items for Systematic Reviews and Meta-analysis (PRISMA) reporting guideline [22]. A protocol for this study is registered with PROSPERO, CRD42024551909. As this study leveraged publicly available data sources, no institutional review board approval was necessary.

### Study design and article selection

We identified studies that examined FT in patients with breast cancer by searching the following electronic databases: MEDLINE (Ovid), Embase (Elsevier), Web of Science Core Collection (Clarivate), PsycINFO (EBSCO), Cumulative Index of Nursing and Allied Health Literature (CINAHL, EBSCO), EconLit (ProQuest), and Sociological Abstracts (ProQuest). The search strategy, which was developed by a medical librarian (PAB) and 2 investigators (SPM, CAM), included terms for breast cancer and for FT or hardship, catastrophic costs or losses, and health care costs among cancer patients and survivors (Supplement). Searches were carried out on June 26, 2024. Appropriate controlled vocabulary terms were included when available. The search was limited to articles published in English between the years 2010 and 2024; a geographic limit for the U.S. was applied in databases where controlled vocabulary was available.

Primary studies of any type that described age as a factor in the analysis of FT related directly to stage I–III breast cancer treatment or indirectly to treatment-related adverse events were included. Treatment-related adverse events of interest included physical complications such as infertility, osteoporosis, cardiac toxicity, pulmonary toxicity, sexual dysfunction, chemotherapy-induced peripheral neuropathy, and breast cancer-related lymphedema [23]. Psychological treatment-related adverse events (i.e., chemotherapy-related cognitive impairment and cancer-related fatigue) were also considered in the search [18]. Given that systemic therapy is a primary driver of financial hardship for women with breast cancer [24], studies were excluded if they included patients with stage 0 (i.e., ductal carcinoma in situ [DCIS]) for whom systemic therapy is not standard. Studies that sampled patients with stage IV breast cancer without sensitivity analyses to distinguish how FT might differ among those with stage I-III cancer were also excluded considering that the predictable and significant financial burden associated with metastatic disease might bias effect size [25]. Additional exclusion criteria included investigations that assessed costs of treatment alone, used qualitative methods alone, were in a language other than English, those that were published prior to 2010 when FT became a widely recognized construct [26], and those that used a composite quality of life metric to assess financial hardship rather than a specific or validated measure of FT. Although many quality of life metrics include financial concerns as a domain of interest, composite scores that aggregate multiple domains into a single index can underestimate the magnitude of financial burden and obscure the true extent of nature of financial distress [27, 28]. Citations for which full text was not available, editorials, reviews, and conference abstracts were also excluded. Relevant articles were screened using Covidence systematic review software (Veritas Health Innovation, Melbourne, Australia).

A data abstraction form was created by 2 authors (SPM, CRM). After removal of duplicate records, titles and abstracts of articles were reviewed independently by 2 investigators (SPM, CRM). Full-text review was completed for manuscripts that were retained after initial screening. Discrepancies were resolved by discussion. A third author (CAM) was available to review records for which agreement could not be reached.

### Data extraction and analysis

Data was abstracted in September 2024. The primary outcome of interest was age-based differences in FT resulting from treatment of stage I-III breast cancer. The secondary outcome of interest was FT due to treatment-related adverse events as classified previously. Study quality was assessed by 2 authors independently (SPM, CRM) using a critical appraisal tool to assess risk of bias [29]. Using the National Heart, Lung, and Blood Institute Quality Assessment Tool for Observational Cohort and Cross-sectional Studies, fourteen criteria were used to assess overall quality rating as good, fair, or poor [30].

## Results

The electronic database search returned 4023 unique records (Fig. 1). Of these, 3957 records were excluded based on the relevance of the title and abstract to the outcomes of interest. Full-text review was performed for 66 articles (Supplemental Table 1). No studies specifically compared the primary outcome of FT using a validated metric that considered FT as a multifaceted construct incorporating psychosocial distress among YA versus non-YA women with stage I-III breast cancer. Thirty were excluded because they addressed costs alone without addressing other domains of FT (i.e., psychological impact and behavioral consequences). Twenty-eight investigations described interventions for FT without data that could clarify the effect of age on financial burden outside of trial circumstances. The majority of articles that were excluded after full-text review included those with noninvasive cancer and/or metastatic disease in their study sample.

Two studies considered age as an independent variable of interest without specifying YA status. These studies both received the highest quality rating (good; Supplemental Table 2). The study by Mazanec et al. analyzed data from 119 women with stage I-III breast cancer collected between 2019 and 2021 as part of a multi-site longitudinal repeated measures mixed-methods investigation: The Symptom Experience, Management and Outcomes According to Race and Social Determinants of Health (SEMOARS model) during Breast Cancer Chemotherapy [31]. The primary outcome of interest was score on The Symptom Distress Scale [32, 33], a 13-item scale validated in a heterogenous group of oncology patients that measures frequency and severity of symptoms. These symptoms include appearance, concentration, bowel function, fatigue, pain, insomnia, appetite alteration, nausea, cough, outlook, and breathing. This investigation considered FT using the Psychological Sense of Economic Hardship scale. The 17 items in the scale assess domains of financial strain (2 items), perception of insufficient resources (2 items), assessment of resources barriers (4 items), and economizing behaviors (9 items). Scales assessing financial strain, perceived and actual resource barriers used a 5-point response scale with a composite score averaged from individual items. Responses to items interrogating economizing behaviors were dichotomized (yes/no). All four domains could be combined to a single score using weight Z-scores for each subscale. Analysis of data demonstrated that younger age was correlated with greater symptom distress (*r* = − 0.20, *p* = 0.027) and that participants with higher symptom distress reported higher levels of economic hardship (*r* = 0.50, *p* < 0.001). In their multivariable analysis of factors associated with symptom distress, younger women and those with economic hardship had higher degrees of symptom distress (coefficient − 0.16, 95% CI 19.95, 43.68, *p* < 0.001 for age; coefficient 0.36, 95% CI 0.34, 1.34, *p* = 0.001).

The second study considered was an analysis of 395 women who participated in a multisite randomized controlled trial investigating breast cancer surgery decision aids. Politi et al. used data collected between 2017 and 2019 prior to surgery and at serial time points until 1 year postoperatively to understand factors associated with out-of-pocket costs and FT using the COmprehensive Score for financial Toxicity (COST) survey [34, 35]. The original measure comprises 12 items, 11 of which are scored. Each item is scored on a 5-point Likert scale (0-Not at all, 4-Very much) with a summated possible score ranging from 0 to 44, with lower values reflecting a greater degree of FT. The scale was validated in patients with stage IV solid tumors (according to the American Joint Committee on Cancer) with a median age of 59 years (range 27–88); 22% of the sample being ≤ 50 years. After consulting the scale developer, the investigators utilized an abbreviated scale with 4 items scored in a similar fashion to the original scale. After adjusting for socioeconomic status, race, surgery type, cancer stage, and study site, compared to women < 65 years, those ≥ 65 years had lower FT at 1 week postoperatively (coeff − 2.58; 95% CI 03.41, − 1.74), 12 weeks postoperatively (coefficient − 2.57; 95% CI − 3.48, − 1.67), and 1 year post surgery (coefficient − 3.57; 95% CI − 5.08, − 2.05).

## Discussion

In completing this systematic review, we found that no studies specifically compared the primary outcome of FT among YA versus non-YA women with resectable invasive breast cancer. Two studies, one of which considered age as a continuous variable and the other which dichotomized age as < 65 years versus 65 years and older, both showed that younger age was associated with increased FT. Although no investigations sought to characterize how treatment-related adverse events exacerbated the association between age and FT, one article did indicate that patients with symptom distress experienced greater financial hardship.

While a minority of breast cancer diagnoses are made in women younger than 40 years, these women suffer worse survival outcomes than women ≥ 40 years at diagnosis [36]. In addition to aggressive tumor biology and advanced disease at presentation among YAs, socioenvironmental factors such as FT may also contribute to this “YA gap” [37] in survival. Prior research suggests those who experience significant financial hardship more frequently adopt maladaptive behaviors such as nonadherence to recommended treatments [38] or oncology appointments [39] to offset costs. These cost-saving strategies, in turn, may compromise health outcomes.

Although the NCCN defines YAs with breast cancer as those who are diagnosed before age 40, individual institutions extend YA programming to those who develop early onset breast cancer before age 45 [40] or 50 [41]. In highlighting the paucity of studies that use the NCCN definition as a threshold for age-based outcomes of interest, this systematic review underscores a lack of consensus on age cutoffs and calls for standardized criteria to improve surveillance and care of early onset cancers [42–44]. Unfortunately, the broad age range included in most studies on FT makes it difficult to discern which direct and indirect costs are most significant for YAs. As an example, since YAs with breast cancer more often present with tumor molecular subtypes and disease stages which require systemic treatment (e.g., chemotherapy), a primary driver of cancer-related costs [45], studies that include DCIS for which systemic therapy is not generally indicated may either skew data toward an older population of women or present findings that dilute our knowledge of FT in the YA population. Additionally, many of the metrics used to assess FT do not address material, psychosocial, or behavioral considerations that are germane to the unique developmental challenges faced by YAs [7, 46]. In their cognitive interviews with adolescents and young adults with cancer and their caregivers, Salsman et al. found that 50% (11/22) of patient participants reported that prioritized items from 73 existing measures of FT were irrelevant. Moreover, the lasting economic consequences of cancer treatment [11, 47] may be more severe among YAs with long life expectancy. In their article, Politi et al. raise the importance of evaluating financial hardship over time by showing that compared to their older counterparts, younger patients had higher odds of financial hardship at 1 year postoperatively than earlier time points. Baseline risk for financial hardship may also vary by age in women under 50; U.S. census data indicates that those in their 20 s to early 30 s earn less per year compared to those ages 30–50 [48]. Our systematic review, which failed to identify studies that compared FT among YA and non-YA women with invasive resectable breast cancer, reflects these issues and identifies a significant gap in knowledge that restricts our ability to create or evaluate YA-specific interventions to reduce financial hardship.

In the current era of personalized medicine and delivery of value-based care, designing interventions to address FT for YAs requires a tailored approach. Financial navigation, a structured method of screening, comprehensive assessment of risk factors, and longitudinal evaluation of outcomes, has shown promise in reducing FT [49]. In a recent study discussing implementation of a financial navigation program, Wheeler et al. demonstrate improvement in COST scores pre and post-intervention among a diverse cohort of oncology patients [50]. While the study includes a high proportion of young patients (18/46 (39%) who participated were younger than 45 years), further studies are needed to shed light on how age and disease subtype affect screening for FT and the efficacy of financial navigation.

Another promising intervention that may be particularly effective in YAs is education to increase health insurance literacy. Qualitative research investigating barriers to care among YAs highlights the importance of understanding coverage for treatments [51, 52]. In their multi-site randomized controlled trial comparing usual financial navigation or tailored health insurance education for 86 patients with newly diagnosed YA cancer, Mann et al. demonstrated improvement in health insurance literacy, awareness of Affordable Care Act protections, and perceived stress [53]. Although COST scores were not significantly different among those who received the interventions compared to those who engaged in usual care navigation, this may again reflect the need for an age-specific metric to assess FT in YAs. Furthermore, as is the case with many well-designed patient-reported outcome metrics, existing measures of FT, including COST, do not have established or validated thresholds to indicate a clinically meaningful difference by which to evaluate efficacy of an intervention [54]. As there is also no standardized metric by which to evaluate improvement in FT, some interventions such as those provided by non-profit organizations that address non-medical costs that are critical for young women during and after treatment (childcare, transportation, food security, etc.) may be overlooked because of lack of longitudinal data demonstrating efficacy.

Given the lasting economic effects documented in these articles, our group has begun to investigate the impact of treatment-related adverse events on perpetuating or exacerbating FT. While no studies in this analysis address this, in their survey-based study of 830 YA women with breast cancer, Tangka et al. found that nearly half of respondents reported taking unpaid time off and 22% endorsed quitting or losing their jobs as a direct result of breast cancer or treatment-related adverse events [55]. Adverse events that develop into chronic disabling conditions, such as breast cancer-related lymphedema and peripheral neuropathy, are not only a source of ongoing spending but also perpetuate FT through work disruption [56]. While the mechanisms by which treatment-related adverse events lead to protracted financial hardship require further investigation, arm morbidity has been shown to predict trajectories of sustained financial difficulty in YAs with breast cancer [57]. The dearth of studies investigating the relationship between treatment-related adverse events and FT represents an opportunity for future exploration. Specifically, care pathways aimed at preventing treatment-related adverse events, e.g., prospective surveillance and early intervention models for breast cancer-related lymphedema [58], could be a novel strategy for reducing long-term financial hardship.

Our study has important limitations. This review focused on validated patient-reported outcome metrics to understand the relationship between age and FT. As such, we may have failed to capture studies which used non-validated FT measures. Additionally, we chose to exclude patients with preinvasive and metastatic breast cancers, thereby restricting the number of studies in our analysis. We acknowledge that women with DCIS having surgical treatment, especially those who experience complications related to post-mastectomy breast reconstruction, and women with stage IV disease are not immune to FT. In fact, the impact of FT would likely have been more significant if these subgroups had been included.

## Conclusion

In this systematic review of over 4000 manuscripts related to breast cancer and FT, we found limited studies that focused on the unique experience of YA patients. Of those eligible for inclusion, our review confirms prior literature, which suggests that younger patients are at increased risk of FT compared to older adult patients. There was insufficient evidence to explore the relationship between treatment-related adverse outcomes and FT. Further work should aim to better understand the unique driving components of FT in YA patients and efforts that mitigate this disparity.

## Supplementary Material

Supplement File 1

**Supplementary Information** The online version contains supplementary material available at https://doi.org/10.1007/s11764-025-01881-7.

## Figures and Tables

**Fig. 1:**
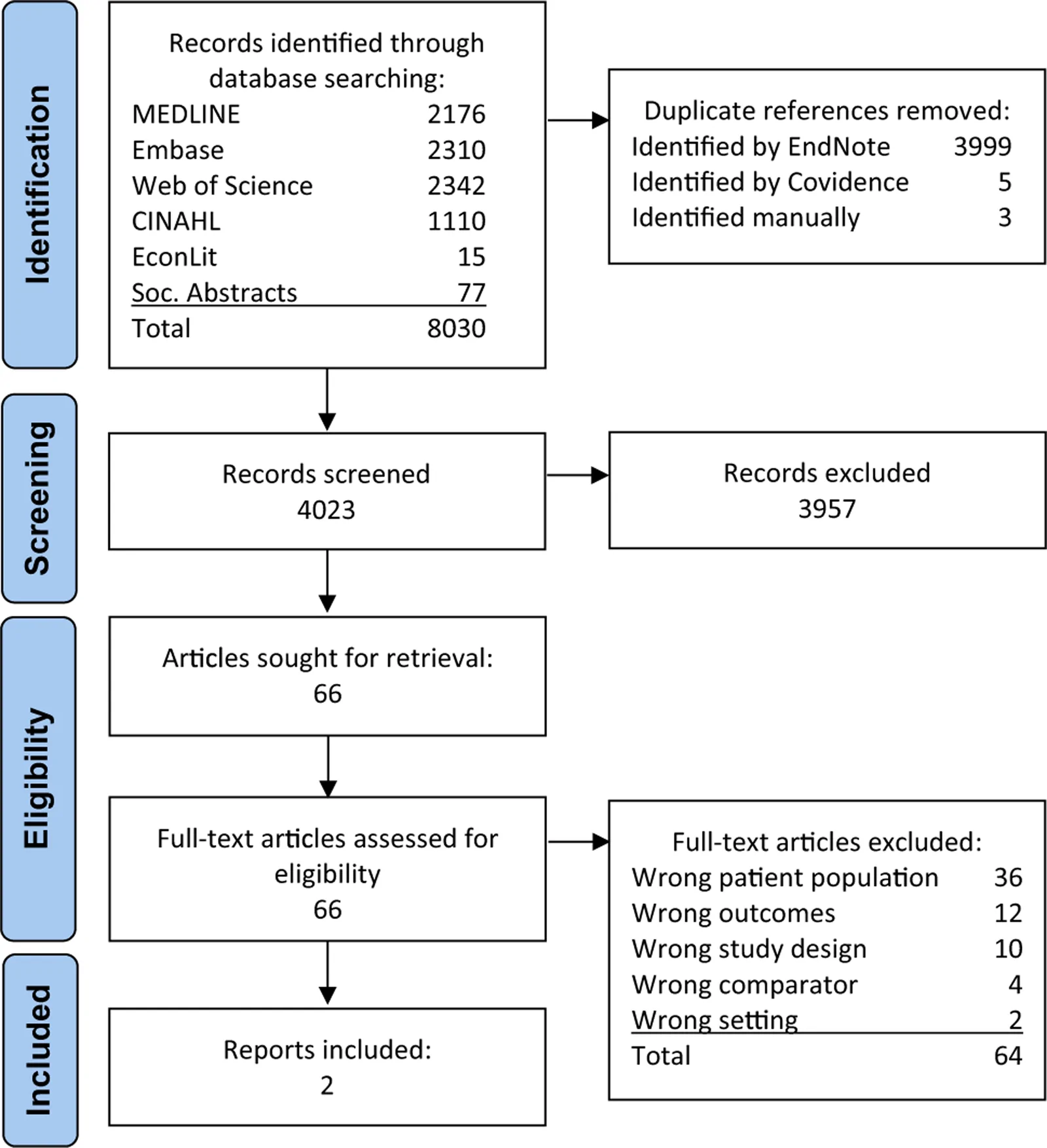
Flow diagram of included studies

## Data Availability

Relevant data will be shared upon written request submitted to Sara Myers.
