# Supplementary material for: Impact of age and treatment-related adverse events on financial toxicity among patients with breast cancer: a systematic review
Source: J Cancer Surviv. Author manuscript; Available in PMC 2026 Nov 3. (PMC13723641; doi:10.1007/s11764-025-01881-7)
Supplement: Supplementary file 1 [file NIHMS2207583-supplement-Supplement_File_1.docx]

**SUPPLEMENTARY MATERIAL**

**Impact of age and treatment-related adverse events on financial toxicity among patients with breast cancer: a systematic review**

**Electronic Database Searches**

MEDLINE (Ovid)

Ovid MEDLINE(R) and Epub Ahead of Print, In-Process, In-Data-Review & Other Non-Indexed Citations, Daily and Versions <1946 to June 21, 2024>

June 26, 2024

2176 Records

1.	breast neoplasms/ or carcinoma, lobular/ or carcinoma, ductal, breast/ or (((breast? or mammary) adj5 (cancer* or tumo?r? or neoplam? or carcinoma? or oncolog*)) or lobular carcinoma?).ab,kf,kw,ti.	513286

2.	exp financial stress/ or (((financial or economic) adj3 (toxicit* or stress or stresses or hardship? or pressure? or strain? or distress or burden? or impact? or difficulty or difficulties or worry or worries or anxiet* or harm? or challenge? or barrier?)) or out-of-pocket).ab,kf,kw,ti.	81956

3.	((cost? adj3 (fear? or barrier? or access*)) or ((catastrophic health or catastrophic healthcare or catastrophic) adj3 (cost? or expense? or expenditure)) or ((income or annual income or wage? or productivity) adj3 (loss or losses or losing or lost)) or ((worry or worries) adj3 afford*) or "effect? on financ*" or ((direct or indirect) adj cost?)).ab,kf,kw,ti.	32126

4.	health care costs/ or (((treatment? or surgery or surgeries or surgical) adj3 cost?) or ((health or health care or healthcare) adj3 (cost? or expenditure? or expense?))).ab,kf,kw,ti.	139414

5.	exp patients/ or exp cancer survivors/ or exp women/ or (patient? or wom?n or survivor?).ab,kf,kw,ti.	9490824

6.	1 and (2 or 3 or (4 and 5))	3767

7.	6 not (comment or editorial or letter or news or newspaper article).pt.	3730

8.	7 not ((exp europe/ or exp asia/ or exp oceania/ or exp caribbean region/ or exp central america/ or exp latin america/ or exp south america/ or exp mexico/ or exp canada/ or exp greenland/) not united states/)	2842

9.	8 and english.lg.	2784

10.	limit 9 to yr="2010-2024"	2176

Embase (Elsevier, 1974 -)

June 26, 2024

2310 Records

1.	'breast cancer'/de OR 'breast carcinoma'/exp OR (((breast$ OR mammary) NEAR/5 (cancer* OR tumor$ OR tumour$ OR neoplam$ OR carcinoma$ OR oncolog*)) OR 'lobular carcinoma$'):ab,kw,ti	685969

2.	'financial stress'/exp OR (((financial OR economic) NEAR/3 (toxicit* OR stress OR stresses OR hardship$ OR pressure$ OR strain$ OR distress OR burden$ OR impact$ OR difficulty OR difficulties OR worry OR worries OR anxiet* OR harm$ OR challenge$ OR barrier$)) OR 'out-of-pocket'):ab,kw,ti	96808

3.	((cost$ NEAR/3 (fear$ OR barrier$ OR access*)) OR (('catastrophic health' OR 'catastrophic healthcare' OR catastrophic) NEAR/3 (cost$ OR expense$ OR expenditure)) OR ((income OR 'annual income' OR wage$ OR productivity) NEAR/3 (loss OR losses OR losing OR lost)) OR ((worry OR worries) NEAR/3 afford*) OR "effect$ on financ*" OR ((direct OR indirect) NEXT/1 cost$)):ab,kw,ti	46708

4.	'health care cost'/de/mj OR 'drug cost'/de/mj OR 'hospitalization cost'/de/mj OR (((treatment$ OR surgery OR surgeries OR surgical) NEAR/3 cost$) OR ((health OR 'health care' OR healthcare) NEAR/3 (cost$ OR expenditure$ OR expense$))):ab,kw,ti	174737

5.	'patient'/de OR 'cancer patient'/de OR 'surgical patient'/de OR 'female'/de OR (patient$ OR woman OR women OR survivor$):ab,kw,ti	15044357

6.	#1 AND (#2 OR #3 OR (#4 AND #5))	7067

7.	#6 NOT ('conference abstract'/it OR 'conference paper'/it OR 'conference review'/it OR 'editorial'/it OR 'letter'/it)	3965

8.	#7 NOT (('Eastern Hemisphere'/exp OR 'Western Hemisphere'/de OR 'South and Central America'/exp OR 'Canada'/exp OR 'Mexico'/exp OR 'Atlantic islands'/exp) NOT 'United States'/exp)	2996

9.	#8 AND [2010-2024]/py 2364

10.	#9 AND [english]/lim	2310

Web of Science Core Collection (Clarivate)

A&HCI , BKCI-SSH , BKCI-S , CCR-EXPANDED , ESCI , IC , CPCI-SSH , CPCI-S , SCI-EXPANDED , SSCI

June 26, 2024

2342 Records

Exact search="on"

1.	TI=(((breast$ OR mammary) NEAR/4 (cancer* OR tumor$ OR tumour$ OR neoplam$ OR carcinoma$ OR oncolog*)) OR "lobular carcinoma$") OR AB=(((breast$ OR mammary) NEAR/4 (cancer* OR tumor$ OR tumour$ OR neoplam$ OR carcinoma$ OR oncolog*)) OR "lobular carcinoma$") OR AK=(((breast$ OR mammary) NEAR/4 (cancer* OR tumor$ OR tumour$ OR neoplam$ OR carcinoma$ OR oncolog*)) OR "lobular carcinoma$")	559378

2.	TI=(((financial OR economic) NEAR/2 (toxicit* OR stress OR stresses OR hardship$ OR pressure$ OR strain$ OR distress OR burden$ OR impact$ OR difficulty OR difficulties OR worry OR worries OR anxiet* OR harm$ OR challenge$ OR barrier$)) OR "out-of-pocket") OR AB=(((financial OR economic) NEAR/2 (toxicit* OR stress OR stresses OR hardship$ OR pressure$ OR strain$ OR distress OR burden$ OR impact$ OR difficulty OR difficulties OR worry OR worries OR anxiet* OR harm$ OR challenge$ OR barrier$)) OR "out-of-pocket") OR AK=(((financial OR economic) NEAR/2 (toxicit* OR stress OR stresses OR hardship$ OR pressure$ OR strain$ OR distress OR burden$ OR impact$ OR difficulty OR difficulties OR worry OR worries OR anxiet* OR harm$ OR challenge$ OR barrier$)) OR "out-of-pocket")	150222

3.	TI=((cost$ NEAR/2 (fear$ OR barrier$ OR access*)) OR (("catastrophic health" OR "catastrophic healthcare" OR catastrophic) NEAR/2 (cost$ OR expense$ OR expenditure)) OR ((income OR "annual income" OR wage$ OR productivity) NEAR/2 (loss OR losses OR losing OR lost)) OR ((worry OR worries) NEAR/2 afford*) OR "effect$ on financ*" OR ((direct OR indirect) NEAR/0 cost$)) OR AB=((cost$ NEAR/2 (fear$ OR barrier$ OR access*)) OR (("catastrophic health" OR "catastrophic healthcare" OR catastrophic) NEAR/2 (cost$ OR expense$ OR expenditure)) OR ((income OR "annual income" OR wage$ OR productivity) NEAR/2 (loss OR losses OR losing OR lost)) OR ((worry OR worries) NEAR/2 afford*) OR "effect$ on financ*" OR ((direct OR indirect) NEAR/0 cost$)) OR AK=((cost$ NEAR/2 (fear$ OR barrier$ OR access*)) OR (("catastrophic health" OR "catastrophic healthcare" OR catastrophic) NEAR/2 (cost$ OR expense$ OR expenditure)) OR ((income OR "annual income" OR wage$ OR productivity) NEAR/2 (loss OR losses OR losing OR lost)) OR ((worry OR worries) NEAR/2 afford*) OR "effect$ on financ*" OR ((direct OR indirect) NEAR/0 cost$))	45292

4.	TI=(((treatment$ OR surgery OR surgeries OR surgical) NEAR/2 cost$) OR ((health OR "health care" OR healthcare) NEAR/2 (cost$ OR expenditure$ OR expense$))) OR AB=(((treatment$ OR surgery OR surgeries OR surgical) NEAR/2 cost$) OR ((health OR "health care" OR healthcare) NEAR/2 (cost$ OR expenditure$ OR expense$))) OR AK=(((treatment$ OR surgery OR surgeries OR surgical) NEAR/2 cost$) OR ((health OR "health care" OR healthcare) NEAR/2 (cost$ OR expenditure$ OR expense$)))	114331

5.	TI=(patient$ OR woman OR women OR survivor$) OR AB=(patient$ OR woman OR women OR survivor$) OR AK=(patient$ OR woman OR women OR survivor$)	9615829

6.	#1 AND (#2 OR #3 OR (#4 AND #5))	3281

7.	Limit #6 to Timespan: 2010-01-01 to 2024-06-30 (Publication Date)	2684

8.	Exclude Meeting Abstract or Proceeding Paper or Editorial Material or Letter from #7	2385

9.	Limit #8 to English Language	2342

CINAHL Complete (EBSCO)

June 26, 2024

1110 Records

1.	MH ("Breast Neoplasms" OR "Carcinoma, Ductal, Breast") OR TI (((breast# OR mammary) N5 (cancer* OR tumor# OR tumour# OR neoplam# OR carcinoma# OR oncolog*)) OR "lobular carcinoma#") OR AB (((breast# OR mammary) N5 (cancer* OR tumor# OR tumour# OR neoplam# OR carcinoma# OR oncolog*)) OR "lobular carcinoma#")	127302

2.	MH ("Financial Stress") OR TI (((financial OR economic) N3 (toxicit* OR stress OR stresses OR hardship# OR pressure# OR strain# OR distress OR burden# OR impact# OR difficulty OR difficulties OR worry OR worries OR anxiet* OR harm# OR challenge# OR barrier#)) OR "out-of-pocket") OR AB (((financial OR economic) N3 (toxicit* OR stress OR stresses OR hardship# OR pressure# OR strain# OR distress OR burden# OR impact# OR difficulty OR difficulties OR worry OR worries OR anxiet* OR harm# OR challenge# OR barrier#)) OR "out-of-pocket")	32426

3.	TI ((cost# N3 (fear# OR barrier# OR access*)) OR (("catastrophic health" OR "catastrophic healthcare" OR catastrophic) N3 (cost# OR expense# OR expenditure)) OR ((income OR "annual income" OR wage# OR productivity) N3 (loss OR losses OR losing OR lost)) OR ((worry OR worries) N3 afford*) OR "effect# on financ*" OR ((direct OR indirect) W1 cost#)) OR AB ((cost# N3 (fear# OR barrier# OR access*)) OR (("catastrophic health" OR "catastrophic healthcare" OR catastrophic) N3 (cost# OR expense# OR expenditure)) OR ((income OR "annual income" OR wage# OR productivity) N3 (loss OR losses OR losing OR lost)) OR ((worry OR worries) N3 afford*) OR "effect# on financ*" OR ((direct OR indirect) W1 cost#))	14408

4.	MH ("Health Care Costs") OR TI (((treatment# OR surgery OR surgeries OR surgical) N3 cost#) OR ((health OR "health care" OR healthcare) N3 (cost# OR expenditure# OR expense#))) OR AB (((treatment# OR surgery OR surgeries OR surgical) N3 cost#) OR ((health OR "health care" OR healthcare) N3 (cost# OR expenditure# OR expense#)))	99089

5.	MH ("Patients+" OR "Cancer Survivors" OR "Women") OR TI (patient# OR woman OR women OR survivor#) OR AB (patient# OR woman OR women OR survivor#)	2650922

6.	S1 AND (S2 OR S3 OR (S4 AND S5))	1865

7.	S6 NOT MH (("Africa+" OR "Europe+" OR "Asia+" OR "Pacific Islands+" OR "Indian Ocean Islands+" OR "Australia+" OR "Atlantic Islands+" OR "Central America+" OR "South America+" OR "Canada+" OR "Mexico+") NOT "United States+")	1398

8.	Limit S7 to Publication Date 20100101-20241231	1121

9.	Limit S8 to English Language	1110

EconLit (ProQuest)

June 26, 2024

15 Records

1.	title(((breast? OR mammary) NEAR/5 (cancer* OR tumor? OR tumour? OR neoplam? OR carcinoma? OR oncolog*)) OR "lobular carcinoma?") OR abstract(((breast? OR mammary) NEAR/5 (cancer* OR tumor? OR tumour? OR neoplam? OR carcinoma? OR oncolog*)) OR "lobular carcinoma?") 396

2.	title(((financial OR economic) NEAR/3 (toxicit* OR stress OR stresses OR hardship? OR pressure? OR strain? OR distress OR burden? OR impact? OR difficulty OR difficulties OR worry OR worries OR anxiet* OR harm? OR challenge? OR barrier?)) OR "out-of-pocket") OR abstract(((financial OR economic) NEAR/3 (toxicit* OR stress OR stresses OR hardship? OR pressure? OR strain? OR distress OR burden? OR impact? OR difficulty OR difficulties OR worry OR worries OR anxiet* OR harm? OR challenge? OR barrier?)) OR "out-of-pocket")	31817

3.	title((cost? NEAR/3 (fear? OR barrier? OR access*)) OR (("catastrophic health" OR "catastrophic healthcare" OR catastrophic) NEAR/3 (cost? OR expense? OR expenditure)) OR ((income OR "annual income" OR wage? OR productivity) NEAR/3 (loss OR losses OR losing OR lost)) OR ((worry OR worries) NEAR/3 afford*) OR "effect? on financ*" OR ((direct OR indirect) PRE/1 cost?)) OR abstract((cost? NEAR/3 (fear? OR barrier? OR access*)) OR (("catastrophic health" OR "catastrophic healthcare" OR catastrophic) NEAR/3 (cost? OR expense? OR expenditure)) OR ((income OR "annual income" OR wage? OR productivity) NEAR/3 (loss OR losses OR losing OR lost)) OR ((worry OR worries) NEAR/3 afford*) OR "effect? on financ*" OR ((direct OR indirect) PRE/1 cost?))	6199

4.	title(((treatment? OR surgery OR surgeries OR surgical) NEAR/3 cost?) OR ((health OR "health care" OR healthcare) NEAR/3 (cost? OR expenditure? OR expense?))) OR abstract (((treatment? OR surgery OR surgeries OR surgical) NEAR/3 cost?) OR ((health OR "health care" OR healthcare) NEAR/3 (cost? OR expenditure? OR expense?)))	2024

5.	[S1] AND ([S2] OR [S3] OR [S4])	28

6	[S5] limited to 2010-2024	15

Sociological Abstracts (ProQuest)

June 26, 2024

77 Records

1.	MAINSUBJECT.EXACT("Breast cancer") OR title(((breast? OR mammary) NEAR/5 (cancer* OR tumor? OR tumour? OR neoplam? OR carcinoma? OR oncolog*)) OR "lobular carcinoma?") OR abstract(((breast? OR mammary) NEAR/5 (cancer* OR tumor? OR tumour? OR neoplam? OR carcinoma? OR oncolog*)) OR "lobular carcinoma?")	3278

2.	MAINSUBJECT.EXACT("Economic problems") OR title(((financial OR economic) NEAR/3 (toxicit* OR stress OR stresses OR hardship? OR pressure? OR strain? OR distress OR burden? OR impact? OR difficulty OR difficulties OR worry OR worries OR anxiet* OR harm? OR challenge? OR barrier?)) OR "out-of-pocket") OR abstract(((financial OR economic) NEAR/3 (toxicit* OR stress OR stresses OR hardship? OR pressure? OR strain? OR distress OR burden? OR impact? OR difficulty OR difficulties OR worry OR worries OR anxiet* OR harm? OR challenge? OR barrier?)) OR "out-of-pocket")	21682

3.	title((cost? NEAR/3 (fear? OR barrier? OR access*)) OR (("catastrophic health" OR "catastrophic healthcare" OR catastrophic) NEAR/3 (cost? OR expense? OR expenditure)) OR ((income OR "annual income" OR wage? OR productivity) NEAR/3 (loss OR losses OR losing OR lost)) OR ((worry OR worries) NEAR/3 afford*) OR "effect? on financ*" OR ((direct OR indirect) PRE/1 cost?)) OR abstract((cost? NEAR/3 (fear? OR barrier? OR access*)) OR (("catastrophic health" OR "catastrophic healthcare" OR catastrophic) NEAR/3 (cost? OR expense? OR expenditure)) OR ((income OR "annual income" OR wage? OR productivity) NEAR/3 (loss OR losses OR losing OR lost)) OR ((worry OR worries) NEAR/3 afford*) OR "effect? on financ*" OR ((direct OR indirect) PRE/1 cost?))	2838

4.	MAINSUBJECT.EXACT("Health care expenditures") OR title(((treatment? OR surgery OR surgeries OR surgical) NEAR/3 cost?) OR ((health OR "health care" OR healthcare) NEAR/3 (cost? OR expenditure? OR expense?))) OR abstract (((treatment? OR surgery OR surgeries OR surgical) NEAR/3 cost?) OR ((health OR "health care" OR healthcare) NEAR/3 (cost? OR expenditure? OR expense?)))	18826

5.	[S1] AND ([S2] OR [S3] OR [S4])	126

6.	[S5] limited to 2010-2024	77

**Supplemental Table 1.** Studies included in full-text review.

| **Study** | **N** | **Ages included (years)** | **Details of financial toxicity reporting** | **Outcome Measure** | **Adverse event reporting** | **Inclusion/Exclusion status** |
| --- | --- | --- | --- | --- | --- | --- |
| Banegas 2022^1^ | 1277 | Mean 63.5 years ± 13.7 SD | Financial hardship reported as social risk factor | Your Current Life Situation Survey | No | Excluded (multiple histologic subtypes) |
| Gharzai 2021^2^ | 32 | Mean 46 years | Costs determined objective financial burden while subjective distress stemmed from the impact of diagnosis and treatment | - | No | Excluded (interview-based study design) |
| Oshima 2021^3^ | 511 | Median age at diagnosis 50 | BC survivors report significant financial burden related to both direct and indirect costs related to care | 88-item questionnaire of previously validated measures as well as novel pretested questions | No | Excluded (did not use a validated metric for financial toxicity) |
| Greenup 2019^4^ | 607 | Median age 49.6 years (range 26-76) | 28% of patients reported costs influenced surgical decisions and were prioritized over breast preservation or appearance. 35% reported financial burden as a result of their cancer treatment. Bilateral mastectomy was associated with FT. | 88-item questionnaire of previously validated measures as well as novel pretested questions | No | Excluded (included DCIS) |
| Eldredge-Hindy 2020^5^ | 88 | Not reported | Radiation therapy was associated with indirect expenses but did not predict FT | Comprehensive Score for Financial Toxicity (COST), Functional Assessment of Cancer Therapy -G7 | No | Excluded (included DCIS) |
| Semin 2020^6^ | 751 | Mean age 54.3 years | Financial distress increases after diagnosis and is highest during treatment; lower social support is associated with higher distress | 18 item survey, Likert scale 1-5 assessing financial distress | No | Excluded (compared patients before and after diagnosis) |
| Zhao 2021^7^ | 1300 | Mean age ± SD, White patients: 61.6 ± 11.6, Black patients: 63.6 ± 11.9, Other patients: 54.9 ± 12.5 | One third of patients endorsed some financial challenge during COVID-19 with a quarter having difficulty accessing treatment | Adapted Patient-Reported Outcomes, Measurement Information System (PROMIS) items and additional 7 items to measure financial challenges | No | Excluded (included DCIS) |
| Maculaitis 2023^8^ | 669 | Mean age 51.6 years ± SD 11.3 | Early-stage BC patients reported greater FT than metastatic BC patients | COVID Stress Scale, Patient Health Questionnaire-8, Psychological Impact of Cancer subscales, Functional Assessment of Cancer Therapy-Breast (FACT-B), Comprehensive Score for Financial Toxicity (COST) | No | Excluded (included DCIS) |
| Hsu 2013^9^ | 285 | Mean 49.8 years ± SD 8.9 | BC survivors have greater financial impacts reported at long term follow-up than controls. | European Organization for Research and Treatment of Cancer Quality of Life Questionnaire C30 (EORTC QLQ-30) with financial impact subscale, Profile of Mood States (POMS), Psychological Adjustment to Illness Scale (PAIS), Impact of Event Scale (IES), Fatigue Symptom Inventory (FSI), Short Form Health Survey (SF-36) | No | Excluded (did not use a validated metric for financial toxicity) |
| Chebli 2020^10^ | 29 | Providers: 70% of sample < 40-50 years old, Patients:22% <40-50 years old | Lack of knowledge and prioritization were associated with FT. | -- | No | Excluded (interview-based study design) |
| Ko 2023^11^ | 53 | Median age 54 | Black patients with breast cancer report challenges associated with FT | 41 item survey covering health care experiences and interactions, cancer-related symptoms and quality of life, lifestyle and behavioral factors, and survivorship information needs | No | Excluded (interview-based study design) |
| Rivers 2021^12^ | 263 | Mean 60.1 years ± 12.7 SD | Decisional distress is related to but distinct from financial distress | 10-item Consumer Financial Protection Bureau Financial Well-Being Scale | No | Excluded (divided patients based on receipt of oral endocrine therapy) |
| Offodile 2021^13^ | 571 | Mean 58 years ± 12.2 SD | Lower financial distress was associated with supplemental insurance, higher income and credit score while greater distress was associated with work reduction or cessation, increased out of pocket spending, tumor stage, and being employed at the time of diagnosis | 30-item questionnaire using questions developed from the relevant published literature, COST questionnaire | No | Excluded (study design - dichotomized patients based on cost scores, population – included DCIS and stage IV disease) |
| Jones 2022^14^ | 2049 | Mean 68.1 years (range 20-85) | Hourly pay and paid sick leave associated with less worry about healthcare costs. Worry about health care costs predicts anxiety. | Questions from the 2018 NHIS questionnaire | No | Excluded (multiple histologic subtypes) |
| Ferreira 2019^15^ | 4262 | Median 56 (IQR 48-65) | Endocrine therapy was associated with reduced quality of life | EORTC QLQ-C30, Hospital Anxiety and Depression Scale | No | Excluded (did not use a validated metric for financial toxicity) |
| Veenstra 2022^16^ | 3672 | Patients: patients <50 years of age constitute 19% of the sample, 50-64 45%, ≥65 36%, Missing 2% | Patients and caregivers face adverse employment outcomes related to cancer treatment contributing to worse HRQoL and perceptions of financial burden among patients | PROMIS Preference, two questions designed for survey about financial burden | No | Excluded (did not use a validated metric for financial toxicity) |
| Berlin 2022^17^ | 883 | Mean 57.1 years ± 11.2 SD | Reconstruction associated with higher OOP costs and perceived decline in financial status, unless patients were employed. | Adapted questions from the Consumer Bankruptcy Project and prior population studies, adapted questions from the Bureau of Labor Statistics | No | Excluded (included DCIS) |
| Jagsi 2014^18^ | 1502 | Age at Diagnosis % < 46, 26.1% 46-55, 36% 56-67, 36.8% ≥ 65 | Racial and ethnic minority women are more vulnerable to economic decline associated with BC | Not detailed, reported to be measures of financial impact including patient perception if they were worse off financially, total amount paid for out-of-pocket medical expenses related to breast cancer | No | Excluded (included DCIS) |
| Ashing 2018^19^ | 116 | Mean age 43 ± 5.4 SD | FT influences access to care, care quality and survivorship outcomes | FACT-G, adapted from Interpersonal Aspects of Care subscale from Adherence Determinants Questionnaire | No | Excluded (did not use a validated metric for financial toxicity) |
| Hastert 2019^20^ | 988 | Mean age 59.6 years ± 9.1 SD | Financial hardship and limiting care due to costs are both associated with lower HRQOL. | FACT-G, measure described in Shankaran et al 2012 | No | Excluded (patient population – not exclusive to breast cancer) |
| Irwin 2014^21^ | 134 | Median age 61 (range 29-88) | 44% of patients report at least moderate financial distress | Study-specific survey questions, InCharge Financial Distress/Financial Well-Being Scale (IFDFW) | No | Excluded (included stage IV disease) |
| Khan 2023^22^ | 34 | Cryoablation: Mean 70.1 ±10.2 (SD), range 54-77, Resection: 66.5 ± 9.9, 54-89 | Costs of care are related to FT | COST, BREAST-Q | No | Excluded (intervention - patients underwent cryoablation rather than standard of care) |
| Lu 2021^23^ | 2588 | Median 31 (IQR 26-35) | BC survivors report greater financial hardship than the general population and engage in medication nonadherence | Questions developed for the study asking about material and subjective experiences of financial hardship | No | Excluded (multiple histologic subtypes) |
| Ramsey 2016^24^ | 231596 | Mean age 53 | Bankruptcy serves as a risk factor for mortality following cancer diagnosis | Bankruptcy filings | No | Excluded (multiple histologic subtypes) |
| Huang 2018^25^ | 101 | Median age at mastectomy Unilateral Mastectomy: 59, CPM: 46 | Patients report that decision to have or not have CPM was impacted by anticipated costs | FACT-B, Satisfaction with Decision Making Scale, asked patients about anticipated and actual costs associated with surgical procedures | No | Excluded (indication - evaluated decision to have contralateral prophylactic mastectomy) |
| Tran 2022^26^ | 4297 | Young Adults defined as those ≤45 comprised 13.3% of the cohort, older adults defined as those >45 | Young adult patients endorse higher FT; income is protective against FT in older but not younger adults | SupportScreen Tool | No | Excluded (multiple histologic subtypes) |
| Tangka 2020^27^ | 830 | Patients age 35-39 comprise 60.5% of the weighted cohort | BC treatment contributed to financial hardships for half of respondents and led to challenges maintaining employment | 66 item instruments asking about insurance status, out of pocket payments, job-related topics, access to treatment and quality of care | No | Excluded (included DCIS) |
| Asaad 2021^28^ | 647 | Mean 55 years ± 12 (SD) | 35% of patients receiving reconstruction reported higher than expected costs and 18% reported major financial distress following treatment | Questions derived from previously published survey items | No | Excluded (included patients without breast cancer who chose risk reduction surgery) |
| Lôbo 2014^29^ | 145 | Mean 52 years ± 11 (SD) | Treatment causes financial difficulties for most patients | EORTC QLQ-C30, Quality of Life Questionnaire Breast Cancer -23 | No | Excluded (setting - study conducted outside of US) |
| Guzik 2021^30^ | 27 | 25-39 (range) | Young adults with cancer need better education about their condition and treatment related to financial considerations. | - | No | Excluded (interview-based study design) |
| Fitzgerald Jones 2022^31^ | 568 | Mean 56.8 ± 11.3 (SD) | Financial hardship associated with 4.64 odds of lymphatic pain | Financial status questions developed for the survey | No | Excluded (did not use a validated metric for financial toxicity) |
| Ivanauskiene 2014^32^ | 338 | Mean 58.6 ± 12.89 (SD) | Poor financial situation and self-reported financial difficulties predicted impaired quality of life | EORTC QLQ-C30, Hospital Anxiety and Depression Scale | No | Excluded (did not use a validated metric for financial toxicity) |
| Williams 2021^33^ | 220 | Median 58 (IQR 49-66) | BC patients prioritized affordability and maintaining functionality when making treatment decisions | COST | No | Excluded (included DCIS) |
| Boukovalas 2021^34^ | 249 | Mean BCT after matching 61.4 ± 10.6 SD, Mean Mastectomy after matching 60.5 ± SD 12.4 | Choice of BCT or mastectomy was not associated with the risk of FT in early-stage cancer | COST | No | Excluded (included DCIS) |
| Li 2024^35^ | 405 | Patients: mean 49.32 ± 9.69 (SD), Caregivers: Mean age 42.28  ± 11.61 (SD) | FT impacts patient and care partner fear of recurrence | COST | No | Excluded (setting - study conducted outside of US) |
| Roine 2019^36^ | 827 | Mean age 61.7 (range 26-90) | Study describes direct and indirect costs of undergoing treatment | Direct and indirect costs of care | No | Excluded (did not use a validated metric for financial toxicity) |
| Sidey-Gibbons 2021^37^ | 611 | Mean 58 years ± 12 SD | Development of machine learning algorithm to predict FT related to BC treatment | COST | No | Excluded (included DCIS and stage IV disease) |
| Palmer 2018^38^ | 157 | 12.6% 20-50 years old, 22.3% 51-60 years old, 43.9% 61-70 years old, 14.6% 71-80 years old, 5.8% >80 years old | 22% of patients developed financial toxicity while receiving RT | Defined based on loss of income, loss of job, loss of spouse, difficulty pay for meals, rent or mortgage, or transportation | No | Excluded (multiple histologic subtypes) |
| Stump 2013^39^ | 400 | Median 61 (range 27-90) | Even among patients with health insurance, cost concerns are prevalent. Screening may help to identify patients with whom clinicians should initiate conversations around cost concerns. | - | No | Excluded (Interview-based study design) |
| Rohani 2015^40^ | 162 | Mean age 46.1 (9.8 SD) | Sense of coherence mediates the impact of BC on reported financial difficulties | EORTC QLQ-C30, SF-12, Health Index, Sence of Coherence | No | Excluded (did not use a validated metric for financial toxicity) |
| Bahar 2023^41^ | 457 | Median 51 (range 24-84) | FT associated with type of surgery, axillary interventions, and chemotherapy | EORTC QLQ-C30, WORTC-BR23 | No | Excluded (did not use a validated metric for financial toxicity) |
| Proussaloglou 2023^42^ | 265 | Median age 47.5 (range 25-74) | 22.7% of BRCA positive patients delayed or avoided care due to financial concerns | COST, SF-12 | No | Excluded (included patients with pathogenic variants without breast cancer) |
| McDougall 2023^43^ | n/a | n/a | n/a | - | No | Excluded (study design - book chapter) |
| Neilson 2023^44^ | 68 | Young Cohort <45 years: n=19, Middle Cohort 45-62 years: n=39, Elderly Cohort >62 years: n=10 | Many BC patients experience significant financial distress without accessing available resources | Survey questions developed for the study | No | Excluded (included DCIS) |
| Mujahid 2011^45^ | 1111 | Mean 50.8 ± 7.8 (SD) | Latina women were more likely to lose a job while receiving BG treatment and women who lost their job were more likely to experience financial strain | Job loss defined by responses to two questions on patient surveys | No | Excluded (did not use a validated metric for financial toxicity) |
| Lee 2011^46^ | 286 | Mean 46.6 years ± 10 (SD) | Patients with BC report worse financial difficulties than the general population a year after diagnosis | EORTC Quality of Life Questionnaire C30, single item on financial difficulties | No | Excluded (patient population – incudes stage IV disease) |
| Markman 2010^47^ | 1767 | Percentage of Respondents by age category <40 4%, 18% 40-49, 36% 50-59, 29% 60-60, 11% 70-79, 2% >80 | Substantial proportions of patients and families experience considerable distress associated with the cost of cancer care, impacting decisions to receive recommended treatments | Survey questions developed for study | No | Excluded (multiple histologic subtypes) |
| McSweeney 2017^48^ | 93 | Above HADS Threshold: Mean age 57.6 ± 6.3  (SD), Normal HADS Range: mean age 55.0 ± 5.7 (SD) | Financial stress associated with positive depression scores | Hospital Anxiety and Depression Scale | No | Excluded (did not use a validated metric for financial toxicity) |
| Baglien 2024^49^ | 839 | Mean 58.2 ± 10.2 (SD) | FT associated with desire to discuss costs with provider | 33 item questionnaire | No | Excluded (did not use a validated metric for financial toxicity) |
| Tesfaye 2024^50^ | 32 | Median 51 (range 41-65) | FT for patients with BI-ALCL was greater when patients had received radiotherapy or had instability in household finances | COST | No | Excluded (patient population - patients diagnosed with breast implant associated anaplastic large cell lymphoma only) |
| Lee 2024^51^ | 50 | 40% of participants ≤ 40 years old | FT has both short- and long-term impacts for patients | - | No | Excluded (interview-based study design) |
| Sun 2014^52^ | 407 | BCT: mean 52.3 years ± 8.5 (SD), Mastectomy: mean 51.9 ± 8.9 (SD), Mastectomy with Reconstruction: mean 56.3 ± 23.2 (SD) | Women who underwent BCT had lower reported financial difficulty than those in the mastectomy group | EORTC QLQ-30, EORTC Quality of Life Questionnaire breast-cancer specific module, Rosenberg self-esteem scale, beck depression index, body image scale, cancer rehabilitation evaluation system sexual scale | No | Excluded (included DCIS) |
| Wu 2024^53^ | 669 | Mean 51.6 years (range 28-82) | FT more common in patients with metastatic disease, younger patients, and those with lower incomes | COST | No | Excluded (included stage IV disease) |
| Myers 2024^54^ | 1126 | Responders: Mean 51.5 ± 9.8 (SD), Non-responders: Mean 52.8 ± 10 (SD) | 13% of patients had some degree of financial difficulty | EORTC QLQ-C30 | Arm morbidity was associated with increased risk of financial hardship | Excluded (included DCIS) |
| Kayser 2021^55^ | 26 | Not reported | Cancer survivors do not experience FT as a singular process | - | No | Excluded (interview-based study design) |
| Benedict 2022^56^ | 273 | Mean 54.6 ± 12.08 (SD) | Younger patients had worse FT | COST, Patient Health Questionnaire – 4, FACT-G | No | Excluded (multiple histologic subtypes) |
| Bailey 2021^57^ | 350 | Mean 50.7 years ± 10.2 (SD), mean 51.2 ±11.5 (SD), Mean 58.5 ± 10.6 (SD) | No association between type of reconstruction and FT. Supplemental insurance and income associated with lower risk of FT while radiation associated with worsening FT. | Survey developed for study and COST | No | Excluded (comparator - based on reconstruction subtype) |
| Thronicke 2023^58^ | 538 | Median 65 years (IQR 54-72) | Baseline quality of life is associated with oncologic outcomes and emphasizes addressing financial distress prior to treatment | EORTC QLQ-C30 | No | Excluded (multiple histologic subtypes) |
| Borowczak 2021^59^ | 15 | Mean 56.3 ± 10.86 (SD) | Sexual and gender identity is related to financial experience of cancer. | - | No | Excluded (interview-based study design) |
| Beuchemin 2023^60^ | 3500 | Age range 18-39 7.3% of sample, 40-49 13.9%, 50-59 24.6%, 60-69 27%, ≥70 27.3% | Demonstrated feasibility of systematic financial screening | COST | No | Excluded (multiple histologic subtypes) |
| **Politi 2021**^61^ | **395** | **Patients aged <65 58.5% of sample, patients aged ≥65 41.5% of sample** | **Higher out of pocket costs are associated with higher FT 1- and 12-weeks following surgery. Older adults reported lower FT scores than younger adults.** | Four items from the COST measure | No | **Included** |
| **Mazenac 2021**^62^ | **119** | **Mean age 54.97 (range 28-80)** | **Perceived economic hardship contributes to greater symptom distress** | Psychological Sense of Economic Hardship scale | No | **Included** |
| Wheeler 2018^63^ | 1494 | Mean age at diagnoses ± SD, White: 52.96 ± 11.2, Black 51.89 ± 10.9 | 58% of women report a negative financial impact of cancer | Not detailed, questions related to financial impacts of cancer diagnosis | No | Excluded (included stage IV disease) |
| Asaad 2020^64^ | 104 | Unilateral Mastectomy: Mean age 51 ± 11 (SD), CPM: mean age 50 ± 10 (SD) | CPM was not associated with a higher degree of patient reported FT | COST, Breast-Q, Short Form-12 | CPM was associated with higher minor complication rates | Excluded (included patients receiving prophylactic risk reducing surgery) |
| Ganesh-Kumar 2022^65^ | 922 | Mean 58.6 years ± 10.3 (SD) | 23.8% of patients reported FT from reconstruction, younger age was associated with greater risk of FT | Questions designed for the study including self-reported financial toxicity either attributable to or not to breast reconstruction | Patients who had a post-operative complication were more likely to report FT | Excluded (included patients receiving prophylactic risk reducing surgery) |
| Perry 2019^66^ | 309 | Mean 58.31 years ± 11.07 (SD) | 37.5% of patients endorsed financial strain and 26.1% reported treatment related financial toxicity | Fourteen item checklist asking patients to indicate financial strain, single item from the Patient Satisfaction Questionnaire, Depression Anxiety Stress Scale, PROMIS depression and anxiety scales, FACT-G | No | Excluded (included patients with stage IV disease) |

1.	Banegas, M. P. *et al.* Association of Social Risk Factors With Mortality Among US Adults With a New Cancer Diagnosis. *JAMA Netw. Open* **5**, e2233009 (2022).

2.	Gharzai, L. A. *et al.* Financial Toxicity During Breast Cancer Treatment: A Qualitative Analysis to Inform Strategies for Mitigation. *JCO Oncol. Pract.* **17**, e1413–e1423 (2021).

3.	Oshima, S. M. *et al.* Patient Perspectives on the Financial Costs and Burdens of Breast Cancer Surgery. *JCO Oncol. Pract.* **17**, e872–e881 (2021).

4.	Greenup, R. A. *et al.* Financial Costs and Burden Related to Decisions for Breast Cancer Surgery. *J. Oncol. Pract.* **15**, e666–e676 (2019).

5.	Eldredge-Hindy, H. B., Yusuf, M. B., Pan, J. & Rai, S. N. Financial Toxicity (FT) in Patients (pts) with Breast Cancer (BC) Receiving Radiation Therapy (RT): A Prospective Observational Study. *Int. J. Radiat. Oncol.* **108**, e47 (2020).

6.	Semin, J. N., Palm, D., Smith, L. M. & Ruttle, S. Understanding breast cancer survivors’ financial burden and distress after financial assistance. *Support. Care Cancer* **28**, 4241–4248 (2020).

7.	Zhao, F. *et al.* The impact of coronavirus disease 2019 on the quality of life and treatment disruption of patients with breast cancer in a multiethnic cohort. *Cancer* **127**, 4072–4080 (2021).

8.	Maculaitis, M. C. *et al.* Psychosocial Wellbeing among Patients with Breast Cancer during COVID-19. *Curr. Oncol.* **30**, 3886–3900 (2023).

9.	Hsu, T., Ennis, M., Hood, N., Graham, M. & Goodwin, P. J. Quality of Life in Long-Term Breast Cancer Survivors. *J. Clin. Oncol.* **31**, 3540–3548 (2013).

10.	Chebli, P. *et al.* Multilevel determinants of financial toxicity in breast cancer care: perspectives of healthcare professionals and Latina survivors. *Support. Care Cancer* **28**, 3179–3188 (2020).

11.	Ko, N. Y., Fikre, T. G., Buck, A. K., Restrepo, E. & Warner, E. T. Breast cancer survivorship experiences among Black women. *Cancer* **129**, 3087–3101 (2023).

12.	Rivers, A. S. & Sanford, K. A special kind of stress: Assessing feelings of decisional distress for breast cancer treatment decisions. *Patient Educ. Couns.* **104**, 3038–3044 (2021).

13.	Offodile, A. C. *et al.* Financial Toxicity Following Surgical Treatment for Breast Cancer: A Cross-sectional Pilot Study. *Ann. Surg. Oncol.* **28**, 2451–2462 (2021).

14.	Jones, S. M. W. Financial worry in people with cancer: Relationship to employment and outcomes. *Psychooncology.* **31**, 1835–1842 (2022).

15.	Ferreira, A. R. *et al.* Differential impact of endocrine therapy and chemotherapy on quality of life of breast cancer survivors: a prospective patient-reported outcomes analysis. *Ann. Oncol.* **30**, 1784–1795 (2019).

16.	Veenstra, C. M., Braun, T. M., Abrahamse, P. H., Wittmann, D. & Hawley, S. T. Employment outcomes in family supporters of patients with early stage breast cancer and their association with patients’ health-related quality of life and financial burden. *Cancer Med.* **11**, 1324–1335 (2022).

17.	Berlin, N. L. *et al.* Perceived Financial Decline Related to Breast Reconstruction Following Mastectomy in a Diverse Population-Based Cohort. *Cancer* **128**, 1284–1293 (2022).

18.	Jagsi, R. *et al.* Long-Term Financial Burden of Breast Cancer: Experiences of a Diverse Cohort of Survivors Identified Through Population-Based Registries. *J. Clin. Oncol.* **32**, 1269–1276 (2014).

19.	Ashing, K. T., George, M. & Jones, V. Health-related quality of life and care satisfaction outcomes: Informing psychosocial oncology care among Latina and African-American young breast cancer survivors. *Psychooncology.* **27**, 1213–1220 (2018).

20.	Hastert, T. A. *et al.* Financial Hardship and Quality of Life among African American and White Cancer Survivors: The Role of Limiting Care Due to Cost. *Cancer Epidemiol. Biomarkers Prev.* **28**, 1202–1211 (2019).

21.	Irwin, B. *et al.* Patient Experience and Attitudes Toward Addressing the Cost of Breast Cancer Care. *The Oncologist* **19**, 1135–1140 (2014).

22.	Khan, S. Y. *et al.* The Role of Cryoablation in Breast Cancer Beyond the Oncologic Control: COST and Breast-Q Patient-Reported Outcomes. *Ann. Surg. Oncol.* **30**, 1029–1037 (2023).

23.	Lu, A. D. *et al.* Medical Financial Hardship in Survivors of Adolescent and Young Adult Cancer in the United States. *JNCI J. Natl. Cancer Inst.* **113**, 997–1004 (2021).

24.	Ramsey, S. D. *et al.* Financial Insolvency as a Risk Factor for Early Mortality Among Patients With Cancer. *J. Clin. Oncol.* **34**, 980–986 (2016).

25.	Huang, J. & Chagpar, A. Impact of anticipated financial burden on patient decision to undergo contralateral prophylactic mastectomy. *Surgery* **164**, 856–865 (2018).

26.	Tran, T. B. *et al.* Emotional Distress and Financial Toxicity in Younger Adult Patients Undergoing Oncologic Surgery. *Ann. Surg.* **276**, 694 (2022).

27.	Tangka, F. K. L. *et al.* Insurance Coverage, Employment Status, and Financial Well-Being of Young Women Diagnosed with Breast Cancer. *Cancer Epidemiol. Biomarkers Prev.* **29**, 616–624 (2020).

28.	Asaad, M. *et al.* Self-Reported Risk Factors for Financial Distress and Attitudes Regarding Cost Discussions in Cancer Care: A Single-Institution Cross-Sectional Pilot Study of Breast Reconstruction Recipients. *Plast. Reconstr. Surg.* **147**, 587e (2021).

29.	Lôbo, S. A., Fernandes, A. F. C., Almeida, P. C. de, Carvalho, C. M. de L. & Sawada, N. O. Qualidade de vida em mulheres com neoplasias de mama em quimioterapia. *Acta Paul. Enferm.* **27**, 554–559 (2014).

30.	Guzik, P. *et al.* Outcomes That Matter Most to Young Adults Diagnosed with Cancer: A Qualitative Study. *J. Adolesc. Young Adult Oncol.* **10**, 534–539 (2021).

31.	Fitzgerald Jones, K. *et al.* Lymphatic Pain in Breast Cancer Survivors. *Lymphat. Res. Biol.* **20**, 525–532 (2022).

32.	Ivanauskienė, R., Padaiga, Ž., Šimoliūnienė, R., Smailytė, G. & Domeikienė, A. Well-being of newly diagnosed women with breast cancer: which factors matter more? *Support. Care Cancer* **22**, 519–526 (2014).

33.	Williams, C. P. *et al.* Quantifying treatment preferences and their association with financial toxicity in women with breast cancer. *Cancer* **127**, 449–457 (2021).

34.	Boukovalas, S. *et al.* Relationship Between Financial Toxicity and Surgical Treatment for Early-Stage Breast Cancer: A Propensity Score-Matched Comparison of Breast-Conserving Therapy and Mastectomy. *J. Am. Coll. Surg.* **233**, 445 (2021).

35.	Li, H. *et al.* Dyadic effects of financial toxicity and social support on the fear of cancer recurrence in breast cancer patients and caregivers: an actor–partner interdependence mediation model. *BMC Nurs.* **23**, 378 (2024).

36.	Roine, E. *et al.* Costs in Different States of Breast Cancer. *Anticancer Res.* **39**, 353–359 (2019).

37.	Sidey-Gibbons, C. *et al.* Development of Machine Learning Algorithms for the Prediction of Financial Toxicity in Localized Breast Cancer Following Surgical Treatment. *JCO Clin. Cancer Inform.* 338–347 (2021) doi:10.1200/CCI.20.00088.

38.	Palmer, J. D. *et al.* Patients Undergoing Radiation Therapy Are at Risk of Financial Toxicity: A Patient-based Prospective Survey Study. *Int. J. Radiat. Oncol.* **101**, 299–305 (2018).

39.	Stump, T. K. *et al.* Cost Concerns of Patients With Cancer. *J. Oncol. Pract.* **9**, 251–257 (2013).

40.	Rohani, C., Abedi, H.-A., Sundberg, K. & Langius-Eklöf, A. Sense of coherence as a mediator of health-related quality of life dimensions in patients with breast cancer: a longitudinal study with prospective design. *Health Qual. Life Outcomes* **13**, 195 (2015).

41.	Bahar, S., Yucel, B. & Erdis, E. Factors Affecting the Quality of Life in Breast Cancer Patients Undergoing Radiotherapy. *Turk. J. Oncol.* (2023).

42.	Proussaloglou, E. M. *et al.* Financial toxicity in BRCA1 and BRCA2 carriers. *Gynecol. Oncol.* **170**, 160–166 (2023).

43.	McDougall, J. A. *et al.* Financial Hardship, Food Insecurity, and Forgone Medical Care. in *Advancing the Science of Cancer in Latinos: Building Collaboration for Action* (eds Ramirez, A. G. & Trapido, E. J.) (Springer, Cham (CH), 2023).

44.	Neilson, T. *et al.* Financial Toxicity of Breast Cancer Care: The Patient Perspective Through Surveys and Interviews. *J. Surg. Res.* **281**, 122–129 (2023).

45.	Mujahid, M. S. *et al.* Racial/ethnic differences in job loss for women with breast cancer. *J. Cancer Surviv.* **5**, 102–111 (2011).

46.	Lee, E. S. *et al.* Health-Related Quality of Life in Survivors With Breast Cancer 1 Year After Diagnosis Compared With the General Population: A Prospective Cohort Study. *Ann. Surg.* **253**, 101 (2011).

47.	Markman, M. & Luce, R. Impact of the Cost of Cancer Treatment: An Internet-Based Survey. *J. Oncol. Pract.* **6**, 69–73 (2010).

48.	Jane McSweeney, L. *et al.* The impact of mode of presentation on distress in patients with early stage breast cancer. *Ir. J. Med. Sci. 1971 -* **186**, 69–71 (2017).

49.	Baglien, B. D. *et al.* Financial Toxicity in Breast Reconstruction: The Role of the Surgeon-Patient Cost-of-Care Discussion. *Semin. Plast. Surg.* **38**, 39–47 (2024).

50.	Tesfaye, E. A., O’Neill, R. C., McGregor, T. & Clemens, M. W. Financial Toxicity in Breast Implant–Associated Anaplastic Large Cell Lymphoma. *Ann. Plast. Surg.* **92**, 34 (2024).

51.	Lee, S. *et al.* Short-term and long-term financial toxicity from breast cancer treatment: a qualitative study. *Support. Care Cancer* **32**, 1–9 (2024).

52.	Sun, Y. *et al.* Comparison of Quality of Life Based on Surgical Technique in Patients with Breast Cancer. *Jpn. J. Clin. Oncol.* **44**, 22–27 (2014).

53.	Wu, Y. *et al.* Financial Toxicity among Patients with Breast Cancer during the COVID-19 Pandemic in the United States. *Cancers* **16**, 62 (2024).

54.	Myers, S. P., Aviki, E., Sevilimedu, V., Thom, B. & Gemignani, M. L. Financial Toxicity Among Women with Breast Cancer Varies by Age and Race. *Ann. Surg. Oncol.* (2024) doi:10.1245/s10434-024-15895-5.

55.	Kayser, K., Smith, L., Washington, A., Harris, L. M. & Head, B. Living with the financial consequences of cancer: A life course perspective. *J. Psychosoc. Oncol.* **39**, 17–34 (2021).

56.	Benedict, C. *et al.* Greater financial toxicity relates to greater distress and worse quality of life among breast and gynecologic cancer survivors. *Psychooncology.* **31**, 9–20 (2022).

57.	Bailey, C. M. *et al.* Understanding the Relationship between Breast Reconstruction Subtype and Risk of Financial Toxicity: A Single-Institution Pilot Study. *Plast. Reconstr. Surg.* **148**, 1e (2021).

58.	Thronicke, A. *et al.* Self-Reported Baseline Quality of Life Mirrors Treatment-Specific Characteristics of Cancer Patients. *Cancers* **15**, 5763 (2023).

59.	Borowczak, M. *et al.* Comparing Breast Cancer Experiences and Quality of Life between Lesbian and Heterosexual Women. *Cancers* **13**, 4347 (2021).

60.	Beauchemin, M. P. *et al.* Implementation of Systematic Financial Screening in an Outpatient Breast Oncology Setting. *JCO Clin. Cancer Inform.* e2200172 (2023) doi:10.1200/CCI.22.00172.

61.	Politi, M. C. *et al.* Women Who Are Young, Non-White, and with Lower Socioeconomic Status Report Higher Financial Toxicity up to 1 Year After Breast Cancer Surgery: A Mixed-Effects Regression Analysis. *The Oncologist* **26**, e142–e152 (2021).

62.	Mazanec, S. R., Park, S., Connolly, M. C. & Rosenzweig, M. Q. Factors associated with symptom distress in women with breast cancer prior to initiation of chemotherapy. *Appl. Nurs. Res.* **62**, 151515 (2021).

63.	Wheeler, S. B. *et al.* Financial Impact of Breast Cancer in Black Versus White Women. *J. Clin. Oncol.* **36**, 1695–1701 (2018).

64.	Asaad, M. *et al.* Financial toxicity and contralateral prophylactic mastectomy: an analysis using propensity score methods. *Breast Cancer Res. Treat.* **183**, 649–659 (2020).

65.	Ganesh Kumar, N., Berlin, N. L., Hawley, S. T., Jagsi, R. & Momoh, A. O. Financial Toxicity in Breast Reconstruction: A National Survey of Women Who have Undergone Breast Reconstruction After Mastectomy. *Ann. Surg. Oncol.* **29**, 535–544 (2022).

66.	Perry, L. M. *et al.* Financial Strain and Physical and Emotional Quality of Life in Breast Cancer. *J. Pain Symptom Manage.* **58**, 454–459 (2019).

**Supplemental Table 2.** Quality assessment for included studies.

|  | **Mazenac 2021^1^** | | | **Politi 2021^2^** | | |
| --- | --- | --- | --- | --- | --- | --- |
| **Criteria** | Yes | No | Other* | Yes | No | Other |
| Was the research question or objective in this paper clearly stated? | X |  |  | X |  |  |
| Was the study population clearly specified and defined? | X |  |  | X |  |  |
| Was the participation rate of eligible persons at least 50%? |  |  | CD | X |  |  |
| Were all the subjects selected or recruited from the same or similar populations (including the same time period)? Were inclusion and exclusion criteria for being in the study prespecified and applied uniformly to all participants? |  |  | CD | X |  |  |
| Was a sample size justification, power description, or variance and effect estimates provided? | X |  |  | X |  |  |
| For the analysis in this paper, were the exposure(s) of interest measured prior to the outcome(s) being measured? | X |  |  | X |  |  |
| Was the timeframe sufficient so that one could reasonable expect to see an association between exposure and outcome if it existed? |  |  | CD | X |  |  |
| For exposures that can vary in amount or level, did the study examine different levels of the exposure as related to the outcome (e.g., categories of exposure, or exposure measured as continuous variable)? | X |  |  | X |  |  |
| Were the exposure measures (independent variables) clearly defined, valid reliable, and implemented consistently across all study participants? |  | X |  | X |  |  |
| Was the exposure(s) assessed more than once over time? |  | X |  |  | X |  |
| Were the outcome measures (dependent variables) clearly defined, valid, reliable, and implemented consistently across all study participants? | X |  |  | X |  |  |
| Were the outcome assessors blinded to the exposure status of the participants? |  |  | CD |  | X |  |
| Was loss to follow-up after baseline 20% or less? |  |  | CD |  | X |  |
| Were key potential confounding variables measured and adjusted statistically for their impact on the relationship between the exposure(s) and outcome(s)? | X |  |  | X |  |  |

*Other: CD: cannot determine; NA: not applicable; NR: not reported

^1^Mazanec, S. R., Park, S., Connolly, M. C. & Rosenzweig, M. Q. Factors associated with symptom distress in women with breast cancer prior to initiation of chemotherapy. *Appl. Nurs. Res.* **62**, 151515 (2021).

^2^Politi, M. C. *et al.* Women Who Are Young, Non-White, and with Lower Socioeconomic Status Report Higher Financial Toxicity up to 1 Year After Breast Cancer Surgery: A Mixed Effects Regression Analysis. *The Oncologist* **26**, e142–e152 (2021).
